# Multiphysics Modeling of Low-Intensity Pulsed Ultrasound Induced Chemotherapeutic Drug Release from the Surface of Gold Nanoparticles

**DOI:** 10.3390/cancers15020523

**Published:** 2023-01-14

**Authors:** Tyler K. Hornsby, Farshad Moradi Kashkooli, Anshuman Jakhmola, Michael C. Kolios, Jahangir (Jahan) Tavakkoli

**Affiliations:** 1 Department of Physics, Toronto Metropolitan University, Toronto, ON M5B 2K3, Canada; 2 Institute for Biomedical Engineering, Science and Technology (iBEST), Li Ka Shing Knowledge Institute, St. Michael’s Hospital, Toronto, ON M5B 1T8, Canada

**Keywords:** DLVO theory, low-intensity pulsed ultrasound, gold nanoparticles, doxorubicin, ultrasound-triggered drug release, targeted cancer treatment

## Abstract

**Simple Summary:**

In targeted chemotherapy, ultrasound can be used to induce the release of anticancer drugs from nanoparticle drug carriers. Our previous study found that after exposure to low-intensity pulsed ultrasound, doxorubicin was released from gold nanoparticle drug carriers, and aggregation of the gold nanoparticles was observed. Currently, no theoretical model of ultrasound-induced drug release from gold nanoparticles exists in the literature. However, DLVO theory can be used to predict the aggregation of colloidal particles. In this work, DLVO theory was applied to predict whether the release of doxorubicin from gold nanoparticle drug carriers would happen under low-intensity pulsed ultrasound exposure. Attractive van der Waals and repulsive electrostatic potentials were calculated for any gold nanoparticle pair, and the total interaction potential was found before and after ultrasound exposure. A threshold for gold nanoparticle aggregation, which indicates doxorubicin release, was then found.

**Abstract:**

Currently, no numerical model for low-intensity pulsed ultrasound (LIPUS)-triggered anticancer drug release from gold nanoparticle (GNP) drug carriers exists in the literature. In this work, LIPUS-induced doxorubicin (DOX) release from GNPs was achieved in an ex vivo tissue model. Transmission electronic microscopy (TEM) imaging was performed before and after LIPUS exposure, and significant aggregation of the GNPs was observed upon DOX release. Subsequently, GNP surface potential was determined before and after LIPUS-induced DOX release, using a Zetasizer. A numerical model was then created to predict GNP aggregation, and the subsequent DOX release, via combining a thermal field simulation by solving the bioheat transfer equation (in COMSOL) and the Derjaguin, Landau, Verwey, and Overbeek (DLVO) total interaction potential (in MATLAB). The DLVO model was applied to the colloidal DOX-loaded GNPs by summing the attractive van der Waals and electrostatic repulsion interaction potentials for any given GNP pair. DLVO total interaction potential was found before and after LIPUS exposure, and an energy barrier for aggregation was determined. The DLVO interaction potential peak amplitude was found to drop from 1.36 k_B_T to 0.24 k_B_T after LIPUS exposure, translating to an 82.4% decrease in peak amplitude value. It was concluded that the interaction potential energy threshold for GNP aggregation (and, as a result, DOX release) was equal to 0.24 k_B_T.

## 1. Introduction

Currently, the first line of treatment for cancer includes surgical resection, chemotherapy, radiation therapy, or a combination of them, all of which can involve significant adverse side effects, nonspecific targeting, or high-dose radiation [1]. In the administration of conventional chemotherapeutic drugs, healthy cells are often damaged along with the tumor cells, causing toxicity to the patient and limiting the achievable dose given to tumors [1]. Drug-loaded nanocarriers can prove beneficial in addressing the limitations of conventional chemotherapy as they allow the efficient transport of drugs in the bloodstream and give protection from premature drug activation. Additionally, nanocarriers allow the potential for both targeted and controlled anticancer drug delivery, induced at the tumor site by various external stimuli, including ultrasound waves [1]. Furthermore, if ultrasound energy absorption is sufficient to heat the tumor site to the therapeutic hyperthermia regime (temperature range of 41–45 °C), blood flow, perfusion, and spacing in endothelial junctions can be increased, further improving anticancer drug uptake [2,3,4].

In previous work by our group [5,6,7], we have developed an ultrasound-mediated nano-sized drug delivery system that uses a patented low-intensity pulsed ultrasound (LIPUS) device to trigger the release of a conventional chemotherapeutic drug, doxorubicin (DOX), from gold nanoparticle (GNP) drug carriers [5,6,7,8,9]. It was determined that the LIPUS device, with an acoustic power setting of 8.40 W at a 50% duty cycle, was effective for drug delivery applications as it could induce anticancer drug release while heating tissue to the hyperthermia temperature regime [6]. The DOX-loaded GNPs were synthesized using a room-temperature green synthesis method, which stabilized as a red-colored colloidal sol of spherical nanoparticles, with trisodium citrate and DOX non-covalently bound to the particle’s surface [5,10]. GNPs were selected for this work, due to their documented inhibition of cancer cell proliferation and inert nature [11,12,13]. In both myeloma and ovarian cancer cell lines, GNPs have been shown to inhibit cancer cell proliferation [11,12], wherein the anticancer properties of GNPs have been attributed to the inhibition of the heparin-binding growth factor involved in proliferation and angiogenesis [13]. When using DOX-loaded GNPs in an ex vivo tissue model, it was found that after five minutes of LIPUS exposure, DOX was successfully released from the GNP surface [6]. However, the underlying mechanisms behind LIPUS-GNP interactions that lead to drug release are poorly understood [14,15].

Parallel to the research advances in experimental applications for targeted anticancer drug delivery, the use of mathematical and computational models to simulate nano-sized drug delivery systems has also become widespread. Numerical models of drug delivery systems can be used to study the efficacy and ultimately expand our fundamental understanding of drug release, transport, and delivery mechanisms [16,17]. Furthermore, the development of numerical models of drug delivery systems can allow researchers to optimize their drug delivery systems before validation in an in vivo model and, eventually, in human clinical trials [18]. This can prove invaluable when designing anticancer drug delivery systems, such as the DOX delivery system introduced by the authors in [6]. Currently, no established numerical model of LIPUS-induced drug release from GNP drug carriers exists in the literature. However, the established Derjaguin, Landau, Verwey, and Overbeek (DLVO) model states that the total interaction of a colloidal particle, such as the DOX-loaded GNPs used previously by our group, can be obtained by taking into account the attractive van der Waals and electrostatic repulsion interaction potentials [19,20].

The attractive van der Waals interaction potential is the driving force behind colloidal nanoparticle aggregation and arises from interactions between dipoles in atoms within the solution [21]. Briefly, the motion of electrons in any atom causes fluctuating temporary dipoles and can induce a dipole in an adjacent atom, leading to attractive electric van der Waals forces [19]. The repulsive electrostatic interaction potential results from electrostatic interactions between permanent charges for a given nanoparticle pair, leading to repulsion, and is a crucial stabilizing mechanism for colloidal dispersion [19]. Experimentally, the strength of the electric potential can be measured directly as zeta potential [19] and is found to decay exponentially with particle separation over the inverse of the Debye length, commonly defined as the double-layer thickness [19,20]. When using the DLVO model, the total DLVO interaction potential between any given GNP pair can be determined by summation of the attractive van der Waals and repulsive electrostatic interaction potentials as a function of temperature [21,22]. When colloidal GNPs are functionalized with anticancer drugs, the stability of the particles can be significantly altered, leading to a loss in stability and subsequent aggregation. To overcome this, additional stabilizing agents can be added to the compound, a classic example being that of citrate-capped GNPs [21]. The ionic strength of the surrounding medium and elevated temperatures in in vivo applications must also be considered when designing GNP drug carriers, as both parameters significantly affect the repulsive electrostatic interaction potential [20,21].

The DLVO model could be applied to model the LIPUS heating of DOX-loaded colloidal GNPs in an ex vivo tissue sample, as demonstrated by the authors in [6]. Currently, DLVO theory has not previously been used to predict drug release from nanoparticle drug carriers; however, it has been used to predict nanoparticle stability and aggregation for cerium oxide [23], silver [24], and fullerene nanoparticles [25]. Kim et al. have also applied DLVO theory to predict the aggregation of citrate-capped GNPs after adding an aggregation-inducing agent, benzyl mercaptan [26,27]. Here, DLVO total interaction potential was calculated before and after benzyl mercaptan addition. The change in DLVO interaction potential peak amplitude was used to study the loss of colloidal stability [26]. For all samples tested, it was discovered that upon the addition of benzyl mercaptan and the subsequent aggregation, the DLVO total interaction potential peak amplitude was significantly reduced [26]. Since the DLVO model is temperature-dependent, a reliable ultrasound tissue heating simulation model must also be used to simulate the LIPUS acoustic field and heat transfer due to ultrasound energy deposition, leading to DOX release. To this end, finite element analysis techniques can prove beneficial. Currently, finite element analysis models have been extensively developed for the focused [28,29] and non-focused [5,6] ultrasound heating of tissue.

In this work, a multiphysics simulation model of our DOX-loaded GNP anticancer drug delivery system was developed. LIPUS-induced acoustic and thermal fields were simulated in an anticancer drug delivery application, and a parametric study of LIPUS parameters was performed. DLVO theory was then applied in the multiphysics model for the first time to predict anticancer drug release. Attractive van der Waals and repulsive electrostatic interaction potentials were calculated for DOX-loaded GNP drug carriers, before and after LIPUS-induced DOX release, and then the DLVO total interaction potential was calculated. Additionally, the change in GNP surface potential was directly quantified using zeta potential measurements before and after LIPUS exposure. Our study used the change in the DLVO total interaction potential before and after LIPUS-induced DOX release to determine a DLVO potential threshold for DOX release under LIPUS exposure. The method to estimate the potential threshold for DOX release can then be applied to predict DOX release under LIPUS exposure for any given LIPUS and GNP parameters when designing anticancer drug delivery systems of this kind.

## 2. Materials and Methods

### 2.1. DOX-Loaded GNP Synthesis

DOX-loaded GNPs were synthesized using a modified green synthesis method, as described in [10]. This slow reduction process of gold salt by trisodium citrate at room temperature has been extensively studied, explored and characterized, with details presented in other works [30,31,32]. First, 0.5 mL of aqueous trisodium citrate solution (38.8 mM) and 20 μL of aqueous 10 mM DOX solution were mixed and sonicated, followed by adding a 0.5 mL aqueous solution of chloroauric acid (4 mM). At room temperature, gold salt was slowly reduced by citrate; in less than an hour, a red-colored colloidal sol of spherical GNPs was formed, with trisodium citrate and DOX attached non-covalently to the surface of spherical GNPs. Finally, the solution was centrifuged and resuspended in MilliQ water to remove any unreacted reactants. Details of room-temperature slow reduction of gold salt by trisodium citrate are provided in [5,30,31,32]. All gold (III) chloride trihydrate (99.9%) and trisodium citrate dihydrate were purchased from Sigma Aldrich, St. Louis, MO, USA. DOX hydrochloride salt (>99%) was purchased from LC Laboratories, Woburn, MA, USA. MilliQ water (Milli-Q^®^ Integral water purification system, Sigma Aldrich) was used for all experiments.

### 2.2. Ex Vivo LIPUS-Induced DOX Release

The procedure for LIPUS-induced DOX release from ex vivo tissue demonstrated in [5,6] was followed to induce DOX release. All ex vivo samples were prepared from freshly excised porcine muscle tissue and submerged in purified water for 12 h to reduce the air bubble content. A 60 × 73 × 25 mm^3^ ex vivo tissue sample was placed in a 3D-printed plastic sample holder, then a 0.5 mL solution of DOX-loaded GNPS was pipetted into a separate 3D-printed GNP holder (10 × 10 × 5 mm^3^) and sealed with a 0.1-millimeter-thick ultrasound transparent film. The 3D-printed GNP holder was then inserted into the tissue sample to position the DOX-GNP sample at the tissue center, and the combined holders were submerged in a 37 °C acrylic water tank with a water heater for temperature control (Haake DC 10 Thermo Controller 003-2859, ThermoFisher Scientific, Waltham, MA, USA). A calibrated thermocouple was also placed inside the GNP chamber to monitor the temperature with an Omega thermometer (HH309A Four-Channel Data Logger, Omega Engineering, Norwalk, CT, USA).

The LIPUS device used in this study was able to operate at three different power settings (8.40, 3.66, and 1.82 W) and three different duty cycle settings (continuous wave, 50% duty cycle, and 40% duty cycle), translating to 9 different total acoustic power settings, with a pulse repetition frequency of 100 kHz and a fixed 5-minute insonation time [9]. In this study, the LIPUS device was fixed to the top of the water tank and turned on at 8.40 W (50% duty cycle) for 5 min to induce DOX release. After LIPUS exposure, the GNP holder was removed, and the DOX-GNP solution was centrifuged. The supernatant was extracted, then the GNPs were re-suspended in purified water.

### 2.3. GNP Characterization and Zeta Potential Measurements

To study GNP aggregation upon LIPUS exposure, DOX-loaded GNP samples collected before and after LIPUS exposure were imaged using transmission electron microscopy (TEM) (HT7800, Hitachi, Tokyo, Japan) and dark-field hyperspectral microscopy (Enhanced Darkfield system, CytoViva, Auburn, AL, USA). UV-vis spectroscopy was performed with a spectrophotometer (Shimadzu UV-3600 Spectrophotometer Kyoto, Kyoto, Japan). For TEM analysis, 2 µL of GNP solution were taken before and after LIPUS exposure and dried on carbon grids for 2 h before imaging. For darkfield imaging, samples before and after LIPUS exposure were dried on glass slides. ImageJ software (ImageJ 1.53, US National Institutes of Health, Bethesda, MD, USA) was used to determine the average GNP diameter from the TEM images before LIPUS exposure. The surface potential was determined before and after LIPUS exposure via direct zeta potential measurements. Zeta potential was measured using a Zetasizer (Zetasizer Ultra, Malvern Panalytical, Malvern, UK) with zeta potential capillary cells (DTS1070, Malvern Panalytical, Malvern, UK). For all zeta potential measurements, a 100 μL sample of GNP solution was diluted in 1 mL of purified water.

### 2.4. Developing a Numerical DLVO Model

To develop a numerical DLVO model of DOX-loaded GNP stability, COMSOL 6.0 (COMSOL Multiphysics Modeling Software, Stockholm, Sweden) was used as a finite-element analysis platform, combined with MATLAB R2022a (MathWorks, Natick, MA, USA) using MATLAB Livelink. The combined COMSOL and MATLAB model-governing equations are provided in the following sections.

#### 2.4.1. Acoustic and Heat Transfer Models

**Figure 1:**
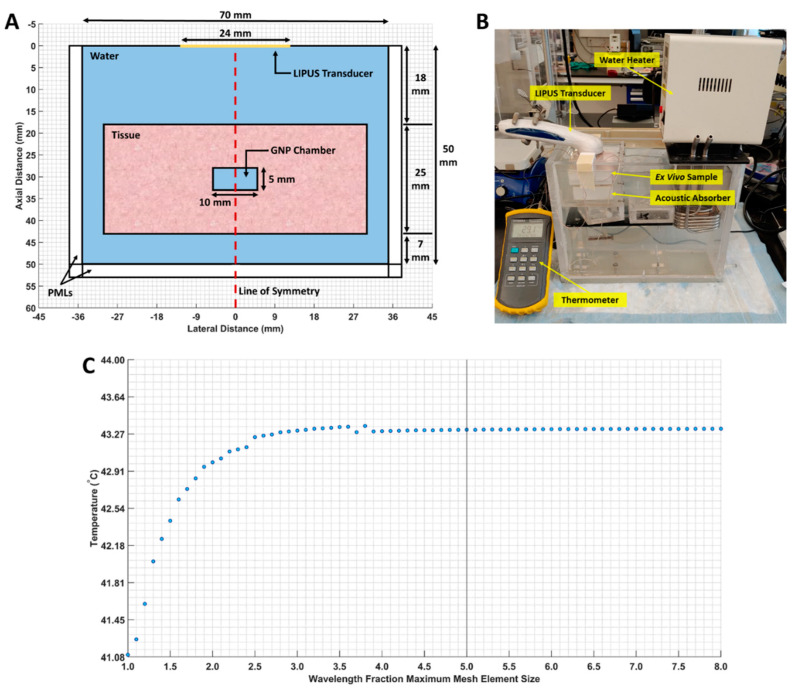
(**A**) The 2D axisymmetric geometry of the computational domain. The water, tissue, GNP chamber (water-filled chamber), and line of symmetry are indicated; (**B**) our ex vivo experimental setup; (**C**) maximum mesh size, versus the average GNP chamber temperature. The selected mesh size is marked by a vertical line.

First, the ex vivo tissue setup outlined in Section 2.2 was simulated as a 2D axisymmetric geometry in COMSOL. A 3-millimeter-wide perfectly matched layer (PML) was applied to the boundaries of the computational domain to prevent LIPUS reflection, and a free triangular mesh was generated throughout the tissue and water domains. A maximum mesh size of (LIPUS wavelength) × (1/5) was selected, based on a parametric sweep examining different mesh sizes. At this maximum mesh size, the average temperature in the GNP chamber did not fluctuate by more than 0.001 °C. The computational domain geometry, our ex vivo experimental setup, and the mesh study are provided in Figure 1.

The LIPUS acoustic field was simulated using COMSOL’s pressure acoustics module in the frequency domain. An inward displacement was applied to the LIPUS transducer face, and the pressure field was simulated by solving the axisymmetric Helmholtz equation, as follows [33]:

(1) $$\frac{\partial }{\partial r}\left[-\frac{r}{\rho }\left(\frac{\partial p}{\partial r}\right)\right]+r\frac{\partial }{\partial z}\left[-\frac{1}{\rho }\left(\frac{\partial p}{\partial z}\right)\right]-{\left(\frac{\omega }{c}\right)}^{2}\frac{rp}{\rho }=0,$$

where p is the acoustic pressure (Pa), ω is the angular frequency (rad/s), ρ is the density (kg/m^3^), c is the speed of sound (m/s), and r and z are the radial and axial coordinates, respectively. An axial symmetry boundary condition was applied at the line of symmetry (r=0), and the PMLs at the bottom and sides of the computational domain were used to prevent reflection. COMSOL’s bioheat transfer (BHT) module was then applied in the time domain to model the LIPUS-induced thermal field. Heat transfer in the water, tissue, and GNP chamber domains was computed by solving Pennes’ BHT equation, as follows [34]:

(2) $$\rho C\frac{\partial T}{\partial t}=k\nabla^{2}T-\omega_{b}C_{b}\left(T-T_{b}\right)+Q_{met}+Q_{ext}$$

in which a subscript of “*b*” denotes that the parameter is for blood and not the domain in question. C is the specific heat capacity (J/(kg∙°C)), T is the temperature (°C), t is time (s), k is thermal conductivity (W/(m∙°C)), $\omega_{b}$ is the blood perfusion rate (kg/(m^3^∙s)), $Q_{met}$ is the metabolic heat generation term (W/m^3^), and $Q_{ext}$ is the external heat source term (W/m^3^). Since we modeled the ex vivo tissue experiments, both $\omega_{b}$ and $Q_{met}$ in Penne’s BHT equation were set to zero [3,35,36]. The external heat source term was equal to the absorbed acoustic energy, to simulate LIPUS absorption at the heat source [33,37], as seen in Equation (3). Note that, here, α is the acoustic absorption coefficient (m^−1^) and I is the time-averaged acoustic intensity (W/m^2^). The $Q_{ext}$ term was also multiplied by a *Rect* function to simulate pulsed ultrasound exposure as a function of time (t). Here, a custom Rect(t) function was used to trigger the LIPUS source as *on* and *off* to simulate pulsing. The off time was changed, based on the desired LIPUS duty cycle (continuous wave, 0.5 ms *on* time, or 0.4 ms *on* time) for a 1 ms period and 5-minute total insonation time.

(3) $$Q_{ext}=\left(2\alpha I\right)Rect\left(t\right)=\left(\frac{\alpha {\left|p\right|}^{2}}{\rho c}\right)Rect\left(t\right)$$

From this, the average temperature in the GNP chamber was found after 5 min of LIPUS exposure and inserted as an input into the MATLAB DLVO model to calculate the interaction potential. The thermal field is assumed to be uniform in the GNP chamber since the computational domain is relatively small and is composed of water. Additionally, the change in the GNP chamber density, specific heat capacity, and thermal conductivity due to the presence of GNPs were not considered [38,39]. This is because an unrealistically high volume-fraction of GNPs in the GNP chamber water volume was needed for a significant change in the calculated DLVO total interaction potential. Key parameters used in the COMSOL simulation are provided in Table 1.

#### 2.4.2. DLVO Model

In the MATLAB Livelink code, both the attractive van der Waals ($V_{vdw}$) and repulsive electrostatic ($V_{elec}$) interaction potentials were calculated for any given GNP pair, before and after LIPUS exposure. The assumption is that upon LIPUS exposure, both the GNP surface temperature and surface potential will change. However, the ionic strength will remain constant since the GNP solution is enclosed inside the 3D-printed GNP chamber, and no volume or reagents are lost. Additionally, a monodispersed sample of GNP drug carriers was assumed. The simplified van der Waals interaction potential for two identical spherical GNPs was used, as follows [19]:

(4) $$V_{vdw}=\frac{-A_{H}}{12}\left[\frac{1}{x\left(x+2\right)}+\frac{1}{{\left(x+1\right)}^{2}}+2ln\frac{x\left(x+2\right)}{{\left(x+1\right)}^{2}}\right]\;\;\;\;\;,\;\;\;\;\;x=\frac{h}{2a}$$

where h is the surface-to-surface separation of the two GNPs (m), a is the GNP radius (m), and $A_{H}$ is the Hamaker constant of GNPs (J). Since the Hamaker constant for GNPs varies in the literature, the average value of 2.5 × 10^−19^ J is typically used [20,26]. The inverse Debye length (κ), used to calculate electrostatic interaction potential, was then found, as follows [42]:

(5) $$\kappa ={\left[\frac{2000e^{2}N_{A}I}{\varepsilon k_{B}T}\right]}^{\frac{1}{2}}$$

where e is the electron charge, $N_{A}$ is Avogadro’s number, I is the ionic strength of the solution (mol/m^3^), $k_{B}$ is the Boltzmann constant, T is the temperature (K), and ε is the temperature-dependent permittivity of water (C^2^/(N∙m^2^)). Note that to determine a value for ε, a temperature-dependent value for the dielectric constant of water was found from [43] and multiplied with the permittivity of the vacuum. I was also taken from the literature as the average ionic strength of citrate-capped GNPs as 0.4052 mol/m^3^ [26]. The electrostatic interaction potential was found using the linear superposition approximation (LSA) for spherical GNPs of equal radius, as follows [42]:

(6) $$V_{elec}=4\pi \varepsilon a^{2}Y^{2}{\left(\frac{k_{B}T}{e}\right)}^{2}\frac{exp\left(-\kappa h\right)}{h+2a}$$

(7) Y=8tanh(eψ04kBT)1+[1−2κa+1(κa+1)2tanh2(eψ04kBT)]12

where $\psi_{0}$ is the GNP surface potential, measured directly as zeta potential (*V*), using a Zetasizer. It is worth mentioning that this model only applies if κa<5 [44]. If κa≥5, the Hogg–Healy–Fuerstenau (HHF) model must be used to find $V_{elec}$ instead [45]. From Equations (4) and (6), the interparticle interaction potentials between two colloidal GNPs were calculated as a function of their surface-to-surface separation and were then used to calculate the DLVO total interaction potential, as follows [19]:

(8) $$V_{DLVO}=V_{vdw}+V_{elec}$$

**Figure 2:**
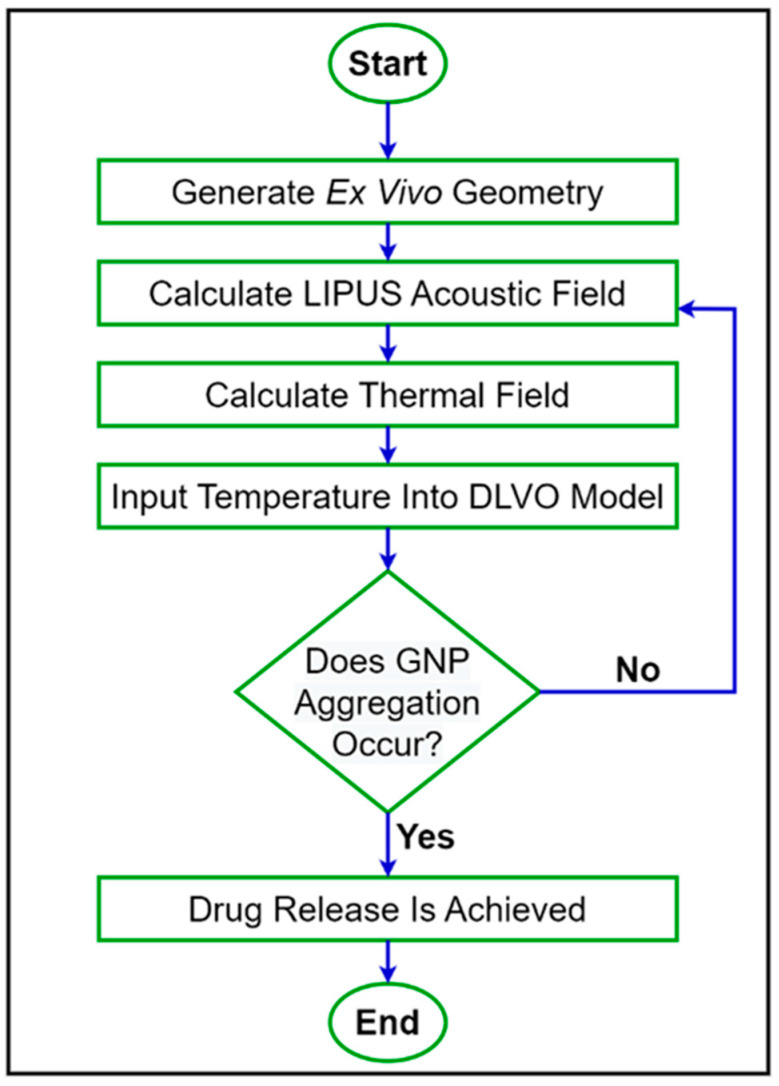
A step-by-step flowchart of the proposed simulation model.

$V_{DLVO}$ was then compared before and after LIPUS exposure to determine the effect on colloidal stability and the potential threshold for GNP aggregation and the subsequent DOX release. A step-by-step flowchart of the proposed simulation model is provided in Figure 2.

Since GNP temperature, diameter, and surface potential are dominant factors in this model, a parametric study of GNP temperature, diameter, and surface potential was performed to assess the effect on DLVO potential. The range of GNP temperature, diameter, and surface potential tested in the parametric study was selected to satisfy the LSA κa<5 condition used in Equation (6). Additionally, all 9 LIPUS total acoustic power settings were input into the model to calculate the DLVO total interaction potential and to study the effect of total acoustic power on DLVO peak amplitude. However, since the LIPUS setting at 8.40 W and at a 50% duty cycle has been shown to induce DOX release from GNP drug carriers in ex vivo tissue, while keeping tissue temperature within the therapeutic hyperthermia temperature regime [6], only this setting was used to induce DOX release experimentally, and this setting was defined as the ideal LIPUS setting. Therefore, the same post-LIPUS exposure GNP surface potential was used for all 9 simulations. The COMSOL-simulated temperature of the GNP solution was also validated by comparing it with thermocouple measurements after LIPUS treatment at 8.40 W at a 50% duty cycle. Lastly, a parametric study of the LIPUS acoustic power and duty cycle on acoustic pressure, time-averaged intensity, and average GNP chamber temperature was performed to study the acoustic and thermal fields generated with all nine possible LIPUS total acoustic power settings. Additionally, this demonstrated the flexibility of the LIPUS acoustic and thermal models and established the relationships between the LIPUS input settings and the acoustic and thermal fields. This also provides a precursor to the parametric study performed with the DLVO model.

## 3. Results

### 3.1. LIPUS-Induced DOX Release in Ex Vivo Tisue Experiments

**Figure 3:**
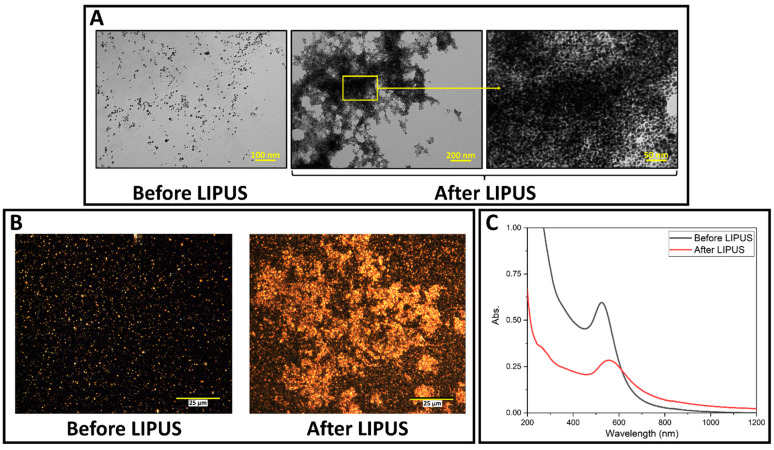
(**A**) TEM images of DOX-loaded GNPs before and after LIPUS exposure in the GNP chamber embedded in ex vivo tissue; (**B**) darkfield microscopy images of DOX-loaded GNPs in the GNP chamber before and after LIPUS exposure in ex vivo tissue. (**C**) UV-vis spectra of DOX-loaded GNPs before and after LIPUS treatment.

LIPUS-induced DOX release was successfully performed in the ex vivo tissue model. GNP samples were collected before and after LIPUS exposure and were characterized by TEM, darkfield microscopy and the UV-vis spectroscopy. GNPs appear as bright spots in the darkfield images, due to their high scattering cross-sections. TEM micrographs, darkfield images, and UV-vis spectra are provided in Figure 3, and show significant GNP aggregation after LIPUS exposure. The UV-vis spectra before LIPUS exposure displays a sharp peak (plasmon band) at 528 nm, suggesting spherical GNPs [30]. After LIPUS treatment, the plasmon band red shifts and broadens due to the aggregation of the nanoparticles. ImageJ particle size analysis was applied to the TEM images before LIPUS exposure, and an average GNP diameter of 7.4 ± 0.5 nm was measured. Zeta potential measurements were also performed before and after LIPUS exposure. Before LIPUS exposure, the average zeta potential was equal to −30.29 ± 1.00 mV; after LIPUS exposure, the average zeta potential was −15.79 ± 2.03 mV. The average zeta potential before LIPUS exposure was averaged over three trials, while the average zeta potential after LIPUS exposure was averaged over six trials. Standard error in both cases was taken as an uncertainty. Lastly, thermocouple measurements were performed in the GNP chamber, which showed that the average final temperature reached at the end of the 5-minute exposure was 43.4 ± 0.3 °C, averaged over 6 trials, with standard error taken as an uncertainty.

### 3.2. LIPUS Heating Simulation Model

**Figure 4:**
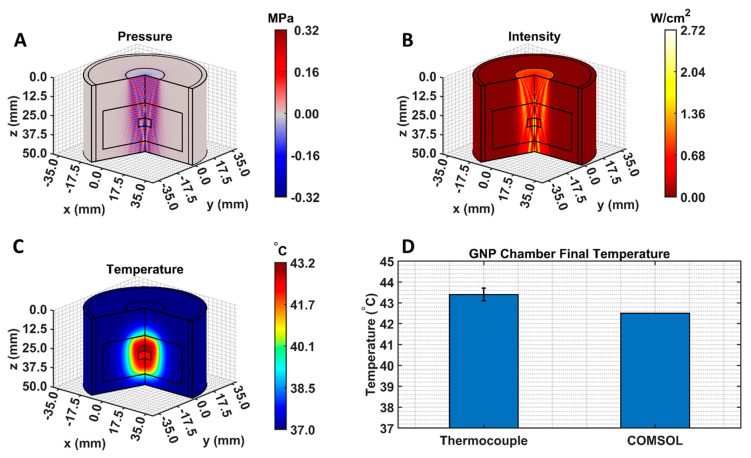
COMSOL-simulated (**A**) pressure, (**B**) intensity, and (**C**) thermal fields. Black lines indicate the LIPUS transducer, porcine tissue domain, and GNP chamber domain. The 2D fields were rotated about the axis of symmetry to produce axisymmetric 3D plots; (**D**) a comparison between the experimental thermocouple measured and the COMSOL-simulated average GNP chamber temperature, reached after 5 min of LIPUS exposure.

**Figure 5:**
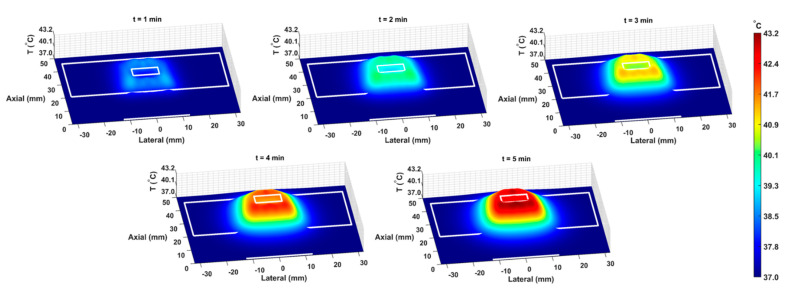
The spatiotemporal thermal field, due to LIPUS heating for the 5-minute LIPUS exposure at 1-minute intervals. White lines indicate the LIPUS transducer, porcine tissue domain, and GNP chamber domain. A height expression was added to the 2D thermal field to show the temperature distribution around the GNP chamber and throughout the tissue sample on the *z*-axis.

**Figure 6:**
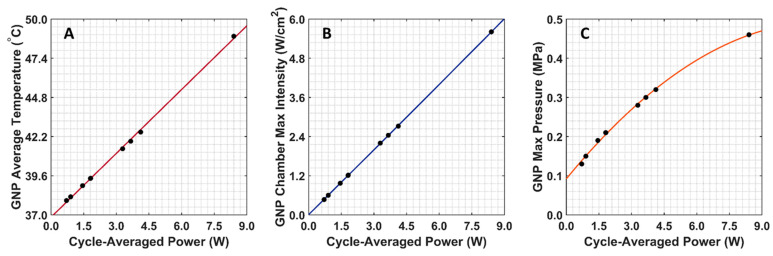
COMSOL-simulated GNP chamber average temperature and time-averaged intensity and pressure for all nine LIPUS total acoustic power settings. (**A**) Average GNP chamber temperature; (**B**) maximum time-averaged intensity in the GNP chamber; (**C**) maximum pressure in the GNP chamber.

The COMSOL-generated acoustic and thermal fields were computed by solving Equations (1) and (2) in the ex vivo tissue geometry and are given in Figure 4A–C. Maximum pressure and intensity were found in the GNP chamber at 0.32 MPa and 2.72 W/cm^2^, respectively. The COMSOL-simulated average temperature in the GNP chamber after 5 min of LIPUS exposure was equal to 42.5 °C. A 2.1% difference between the COMSOL-simulated temperature and thermocouple-measured temperature in the GNP chamber after 5 min of LIPUS exposure was observed (Figure 4D). Spatiotemporal 3D plots of the LIPUS thermal field in the ex vivo tissue sample are also provided in Figure 5, to visualize the heat distribution around the GNP chamber. Here, a maximum tissue temperature of 43.2 °C was reached between the GNP chamber and the tissue edge; however, the average temperature in the GNP chamber was slightly lower, due to the slower heat transfer across the tissue–water boundary. The LIPUS parametric study results show the maximum time-averaged pressure, intensity, and average temperature in the GNP chamber for all nine LIPUS total acoustic power settings (Figure 6). Here, a first-order fit was applied to the GNP chamber average temperature and the time-averaged intensity results, while a quadratic fit was applied to the GNP chamber’s maximum pressure results.

### 3.3. DLVO Model

**Figure 7:**
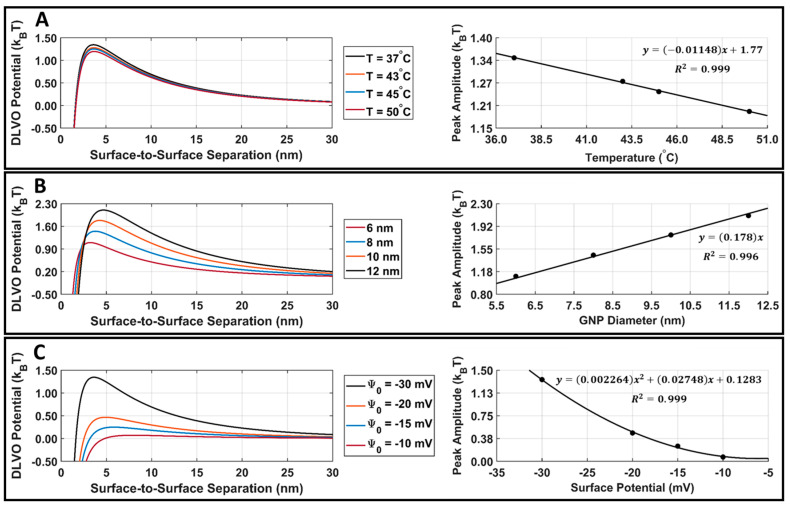
DLVO total interaction potential parametric study results for DOX-loaded GNPs. (**A**) DOX-loaded GNP surface potential is fixed at −30.3 mV, the diameter is fixed at 7.4 nm, and the temperature is varied. This surface potential and diameter were selected as they are the surface potential and diameter of our stable DOX-loaded GNPs. A linear fit of DLVO peak amplitude vs. temperature is also provided for reference purposes. (**B**) DOX-loaded GNP surface potential is fixed at −30.3 mV, the temperature is fixed at 37.0 °C, and the diameter is varied. A linear fit of the DLVO peak amplitude vs. diameter is also provided for reference. (**C**) DOX-loaded GNP temperature is fixed at 37.0 °C, the diameter is fixed at 7.4 nm, and the surface potential is varied. A second-order polynomial fit of the DLVO peak amplitude vs. surface potential is also provided for reference. The R^2^ fit value is provided for all cases.

Attractive van der Waals and repulsive electrostatic potentials were calculated in MATLAB using Equations (4) and (6), and the DLVO total interaction potential energy was found for DOX-loaded GNPs. The GNP temperature, diameter, and surface potential parametric study results are presented in Figure 7. When increasing the GNP temperature or GNP surface potential, there was a decrease in the DLVO peak amplitude (Figure 7A,C). When increasing the GNP diameter, there was an increase in the DLVO peak amplitude (Figure 7B). The DLVO total interaction potential peak amplitude was plotted with respect to GNP temperature, diameter, and surface potential, and curve fitting was applied to demonstrate the relationship between these parameters and DLVO peak amplitude. DLVO peak amplitude was found to follow a linear relationship with GNP temperature and size, along with a second-order polynomial relationship with GNP surface potential.

**Figure 8:**
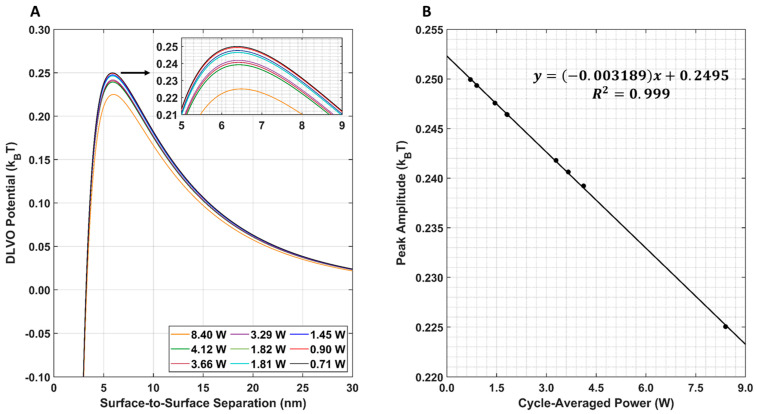
(**A**) DLVO total interaction potential for all nine LIPUS settings. An inset shows peak differences; (**B**) DLVO total interaction potential maximum peak amplitude for all nine LIPUS settings. A linear fit was applied to the data. The R^2^ value of the fit is also provided for reference.

The resulting DLVO total interaction potential for all nine LIPUS settings is provided in Figure 8A. Note that, here, the post-LIPUS treatment GNP surface potential of −15.79 mV was used for all nine simulations. This value was experimentally measured using the 8.4 W at 50% duty cycle LIPUS setting. A 1D plot of the maximum DLVO peak value vs. cycle-averaged power is also provided in Figure 8B. Here, as the cycle-averaged power increases, there is a linear decrease in DLVO peak amplitude.

**Figure 9:**
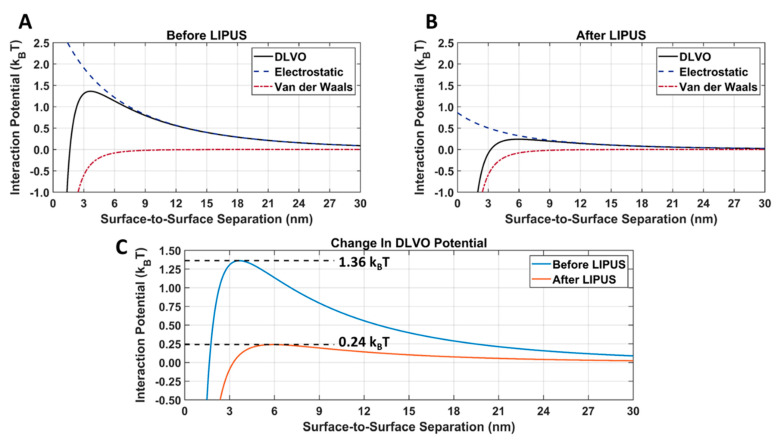
Attractive van der Waals and repulsive electrostatic DLVO total interaction potentials for DOX-loaded GNPs (**A**) before and (**B**) after LIPUS exposure; (**C**) a direct comparison of the DLVO total interaction potential before and after LIPUS exposure.

The van der Waals, electrostatic, and DLVO interaction potentials for DOX-loaded GNPs before and after LIPUS exposure, using the LIPUS setting of 8.4 W at a 50% duty cycle, are provided in Figure 9A. A comparison of the DLVO total interaction potential before and after LIPUS exposure is provided in Figure 9B, and the DLVO parameters used in the calculation are provided in Table 2. In Figure 9A,B an energy barrier for aggregation was evident before and after the LIPUS potential curves. The DLVO peak amplitude decreased from 1.36 k_B_T to 0.24 k_B_T after LIPUS treatment, translating to an 82.35% decrease in amplitude (Figure 9C).

## 4. Discussion

Before LIPUS exposure, a relatively monodispersed population of DOX-loaded GNPs was observed (Figure 3). After LIPUS exposure, significant aggregation was seen in the TEM micrographs and darkfield images. This was further supported by the change in UV-vis spectra after LIPUS treatment. The large-scale aggregation of GNPs suggests that both the trisodium citrate and DOX, bound electrostatically to the GNP surface, were successfully released under LIPUS exposure, as both molecules act as stabilizing agents to keep GNPs spherical in the synthesis [5]. Therefore, GNP aggregation is an indirect indication of DOX release. Furthermore, the significant change in GNP zeta potential (47.9%) observed after LIPUS exposure supports the interpretation of the release of DOX from the GNP surface. Since GNP aggregation occurs with DOX release, the proposed DLVO model can be used to predict the potential threshold for aggregation and, therefore, predict DOX release for any given GNP parameters.

The BHT model can simulate the GNP solution temperatures due to LIPUS heating (2.1% error), compared to the experimental thermocouple measurements (Figure 4). The temperature distribution around the GNP chamber in Figure 5 also shows that the LIPUS transducer can successfully heat the ex vivo tissue sample to the hyperthermia temperature regime, with a maximum temperature of 43.2 °C after 5 min of exposure. The lower average temperature of 42.5 °C after 5 min of exposure in the GNP solution results from the low ultrasound attenuation coefficient of water, as the LIPUS transducer cannot heat water. The heating mechanism in the GNP chamber is probably due to the heat transfer across the ex vivo tissue–water boundary at the membrane of the GNP chamber, due to conduction. Here, we have assumed that there is no fluid motion within the GNP chamber and negligible heat transfer via convection across the ex vivo tissue–water boundary. The LIPUS parametric study (Figure 6) also illustrates the importance of the LIPUS total acoustic power in targeted DOX release. As the total acoustic power increases, the GNP chamber’s average temperature, maximum time-averaged intensity, and maximum pressure also increase. The linear trend of increasing temperature and intensity with cycle-averaged power was observed because the time-averaged intensity is a measurement of power/area and the heat source term in Equation 2 is dependent upon intensity. Similarly, time-averaged intensity is proportional to the maximum pressure squared, leading to a quadratic relationship. Ideally, the LIPUS power should be selected to ensure that the GNP solution temperature remains within the hyperthermia temperature regime, to take advantage of the added anticancer benefits.

The parametric study results of the DLVO model show that the model has a high sensitivity to DOX-loaded GNP temperature, diameter, and surface potential (Figure 7A–C). The DLVO total interaction potential peak amplitude displayed a linear relationship with GNP temperature and diameter; however, a second-order polynomial relationship was observed with the GNP surface potential. This suggests that the DLVO model and GNP stability are highly sensitive to GNP zeta potential. The model also showed higher sensitivity to changes in the GNP surface potential than to temperature with the range of values evaluated. This is likely to be because the range of surface potentials tested was larger than the temperature range; however, no temperatures above 50 °C were included in this study, as this is above the practical LIPUS heating temperature for anticancer drug release and it causes thermal damage to the tissue [2]. Additionally, the range of GNP diameters tested in the parametric study was kept limited, to satisfy the needs of the LSA method used in Equation (6). When comparing the DLVO total interaction potential peak amplitude and surface potential, a second-order polynomial relationship was found. When testing all nine of the cycle-averaged powers available with the LIPUS transducer, as seen in Figure 8, a decrease in the DLVO total interaction peak amplitude, or the energy barrier for aggregation, was also observed due to the changes in GNP chamber temperature. The decrease in DLVO peak amplitude followed a linear relationship with LIPUS cycle-averaged power. The GNP surface potential also has a significant impact on DLVO potential. However, only the post-LIPUS surface potential for 8.40 W and a 50% duty cycle LIPUS setting was used, which is the baseline state of our study. The DLVO maximum peak amplitude, or potential barrier for aggregation, was measured for all nine LIPUS settings, and a linear relationship with LIPUS cycle-averaged power was found. This suggests that as cycle-averaged power increases, the GNP maximum temperature also increases, and colloidal stability decreases. Overall, the GNP temperature after ultrasound treatment primarily depends on the ultrasound modality used to induce anticancer drug release; however, the GNP surface potential highly depends on the synthesis protocol and anticancer drug payload. If this model is used for other anticancer drugs in the future, the GNP surface potential before and after LIPUS exposure could differ, leading to a different DLVO peak amplitude in both cases.

After inputting exposure and zeta potential before and after LIPUS exposure into the DLVO MATLAB Livelink model, a dramatic change in the electrostatic interaction potential was observed (Figure 9). When comparing Figure 9A,B, there was no change in the attractive van der Waals interaction potential, as Equation (4) only depends on the GNP radius. Since the GNP radius is measured from the GNP core, there is no change before and after LIPUS exposure. However, since Equation (6) depends on the GNP surface temperature and surface potential, the electrostatic interaction potential decreased after LIPUS exposure. It is important to note that before LIPUS treatment, the GNPs are in a stable colloidal form and possess the same surface charge, due to the presence of the citrate layer. The mutual forces of repulsion between the GNPs prevent them from aggregating and settling under the action of gravity. Although the electrostatic repulsion potential was decreased after LIPUS exposure, it is still present as a non-zero repulsive potential between each GNP-GNP pair. When comparing the DLVO total interaction potential before and after LIPUS exposure, as seen in Figure 6, there was an approximately 82.4% loss in peak amplitude after LIPUS exposure. This suggests a significant decrease in colloidal stability and depicts a similar trend in the changes in DLVO interaction potential seen in [26], with the addition of an aggregation-inducing agent. Since GNP aggregation and subsequent DOX release were confirmed as occurring at the end of the LIPUS exposure, a DLVO potential threshold for GNP aggregation and, therefore, DOX release is estimated at 0.24 k_B_T. Based on these results, for any given DOX-loaded GNP colloidal solution, DOX release will occur if the DLVO total interaction potential is reduced to the 0.24 k_B_T potential threshold or lower. If the DLVO parameters of any given DOX-loaded GNP colloidal solution are known, this model can be applied to predict aggregation and the subsequent DOX release.

One limitation of the developed DLVO model is that it only considers the thermal mechanisms of LIPUS-induced DOX release. In past work by our group [6], we have shown that, under similar LIPUS exposures, non-thermal mechanisms account for a significant amount of LIPUS-induced DOX release from GNP drug carriers. Therefore, a non-thermal release model could also be applied to the existing DLVO model in future work. Additionally, the developed DLVO model operates on the principle that DOX is released, leading to GNP aggregation. If this model is applied to GNP drug carriers loaded with other anticancer drugs, the GNPs would need to aggregate upon releasing their anticancer drug payload. Furthermore, any change in the GNP surface potential due to changes in the synthesis protocol or the loading of other anticancer drugs would need to be considered. In future work, different anticancer drugs and ultrasound modalities could be evaluated with the proposed DLVO model to explore their specific energy barriers for drug release from the carrier. Lastly, the ex vivo setup used in this study assumes that the GNP-DOX volume is kept contained in the GNP chamber, with a constant ionic strength. As a result, the recombination of released DOX and GNP drug carriers may occur. To expand this model into in vivo studies, these two factors must be considered. Furthermore, blood perfusion and interstitial fluid flow in the tumor microvascular network and interstitium must be included and then coupled with the drug transport equations to accurately model DOX transport and in vivo heat transfer [16,17,46,47,48].

## 5. Conclusions

In this work, a numerical model of LIPUS-triggered DOX release was designed and combined with DLVO theory to predict ultrasound-induced DOX release from gold nanoparticles for the first time. LIPUS-induced DOX release from GNP drug carriers was achieved in an ex vivo tissue model. TEM, darkfield microscopy, and UV-vis techniques were used to determine the finding that the GNPs aggregated upon DOX release. Additionally, a change in the GNP zeta potential values upon DOX release was also observed. A finite-element numerical model was developed to model the LIPUS-induced heating of the ex vivo tissue and was combined with a DLVO total interaction potential model to predict GNP colloidal stability before and after LIPUS exposure. This was achieved using the GNP temperature and zeta potential that were present during GNP aggregation and the subsequent DOX release. Our computational model could simulate GNP solution temperature within 2.1% of the measured thermocouple values. It was found that after LIPUS exposure, a significant drop in the DLVO total interaction potential peak, or energy barrier for aggregation, of 82.4% was observed. It was concluded that a DLVO potential for LIPUS-induced DOX-loaded GNP aggregation, and, therefore, drug release, existed at 0.24 k_B_T. This model is the first application of DLVO theory in ultrasound-targeted anticancer nano-drug delivery systems. It can be used to predict the possibility of drug release when designing future LIPUS-targeted anticancer drug delivery systems.

## Figures and Tables

**Table 1:** Key parameters used in the pressure acoustics and BHT simulation. The material parameters were taken from [40,41].

Domain	Parameter	Value	Unit
LIPUS Transducer	Element Displacement	24.944	nm
	Wavelength	1.5206	mm
	Frequency	1.0	MHz
	Power	8.4	W
	Duty Cycle	50%	-
	Pulse Repetition Frequency	100	kHz
	Element Diameter	24	mm
Water	Attenuation Coefficient	0.0022	dB/(cm∙MHz^2^)
	Density	994.23	Kg/m^3^
	Speed of Sound	1520.6	m/s
	Initial Temperature	37.0	°C
Porcine Tissue	Attenuation Coefficient	0.780	dB/(cm∙MHz^2^)
	Density	1090	Kg/m^3^
	Speed of Sound	1600	m/s
	Initial Temperature	37.0	°C

**Table 2:** Temperature-dependent DLVO parameters and constants used in the DLVO model.

Parameter (Unit)	Symbol	Before LIPUS	After LIPUS	Change Percentage
Measured GNP Temperature (°C)	T	37.0	42.5	14.9
Measured GNP Zeta potential (mV)	$\psi_{0}$	−30.3 ± 1.0	−15.8 ± 2.0	47.9
Measured Water Permittivity (10^−9^ [C^2^/(N∙m^2^)]	ε	1.26	1.23	2.2
Calculated DLVO Maximum Peak Amplitude (k_B_T)		1.4	0.24	82.4
**Parameter**	**Symbol**	**Constant Value**		
GNP Hamaker Constant (10^−19^ J) [20]	$A_{H}$	2.5		
Measured GNP Diameter (nm)	a	7.4		
Ionic Strength (mol/m^3^) [26]	I	0.41		

## Data Availability

GNP chamber average temperature measurements and GNP zeta potential measurements before and after LIPUS treatment are provided at Database: Mendeley [49].
